# Big Data in traumatic brain injury; promise and challenges

**DOI:** 10.2217/cnc-2016-0013

**Published:** 2017-07-10

**Authors:** Denes V Agoston, Dianne Langford

**Affiliations:** 1 Department of Anatomy, Physiology & Genetics, Uniformed Services University, Bethesda, MD 20814, USA; 2 Department of Neuroscience, Karolinska Institute, Stockholm, Sweden; 3 Department of Neuroscience, Lewis Katz School of Medicine, Temple University, Philadelphia, PA 19140, USA

**Keywords:** artificial intelligence, big data, big data analytics, machine learning, traumatic brain injury

## Abstract

Traumatic brain injury (TBI) is a spectrum disease of overwhelming complexity, the research of which generates enormous amounts of structured, semi-structured and unstructured data. This resulting big data has tremendous potential to be mined for valuable information regarding the “most complex disease of the most complex organ”. Big data analyses require specialized big data analytics applications, machine learning and artificial intelligence platforms to reveal associations, trends, correlations and patterns not otherwise realized by current analytical approaches. The intersection of potential data sources between experimental TBI and clinical TBI research presents inherent challenges for setting parameters for the generation of common data elements and to mine existing legacy data that would allow highly translatable big data analyses. In order to successfully utilize big data analyses in TBI, we must be willing to accept the messiness of data, collect and store all data and give up causation for correlation. In this context, coupling the big data approach to established clinical and pre-clinical data sources will transform current practices for triage, diagnosis, treatment and prognosis into highly integrated evidence-based patient care.

Big Data (BD) and Big Data Analytics (BDA) are changing our lives significantly. Most of us use Google and Amazon, and it is difficult not to notice how well they can predict our interests, preferences, etcetera. To be able to do so, Google and Amazon needed several things: huge amounts of data, BD and new tools including BDA, Artificial Intelligence (AI) and Machine Learning (ML). These tools are capable of processing and analyzing mountains of data to generate correlations and make predictions. The BD approach thus allows Google, Amazon and others to find correlations, make predictions, and generate new information and knowledge.

One of the most clear beneficiary of using BD and BDA approaches would be the ‘most complex disease of the most complex organ’, traumatic brain injury (TBI). TBI is when ‘physics meets biology’, in other words, when physical forces suddenly disrupt the structural integrity of the brain leading to functional impairments. Approximately 70% of TBI cases are caused by sudden acceleration/deceleration of the head resulting from falls, traffic and sport accidents, among others[1]. Over 85% of TBIs are mild, also called concussion, and result in large part from playing contact sports [2]. The physical forces of impact can be measured, recorded and analyzed in the context of the biological response. Using the BD approach, a TBI ‘dosimetry’ can be established that, in analogy with ionizing radiation, can assess injury severity, guide therapeutic interventions and provide predictions. In the case of severe TBI, modern neurointensive care monitors dozens of physiological and biochemical parameters, thereby generating huge amounts of real-time data. Analyzing such data in the context of outcomes using the BD approach can significantly assist in making therapeutic decisions. Using the BD approach in experimental TBI research would help to close the substantial gap between preclinical and clinical studies by collecting and analyzing the physical data in the context of biological response. The third critically important application of the BD approach is to ‘mine’ existing, legacy data published in the scientific literature. This latter task is probably even more challenging than designing and performing experiments and studies with set parameters in mind such as Common Data Elements (CDEs) and BD approaches.

Despite enormous scientific and monetary investment during the last several decades to identify evidence-based, specific, efficient pharmaco- (or other) therapy, there is still no treatment to mitigate the acute and the long-term consequences of TBI. These facts clearly show that the TBI field has reached its ‘strategic inflection point’ and that repeating the same will not result in new and much needed information and knowledge. The BD approach offers solutions to many of TBI's most vexing issues.

In this review, we will briefly outline the potential of BD and BDA, and the possible benefits and main challenges of using these approaches in experimental and clinical TBI.

## Big Data

BD is a term for extremely large datasets that are so large and complex that they cannot be analyzed using traditional data processing applications [3–15]. The analysis of BD requires specialized BDA, AI and ML that can reveal patterns, trends, associations, correlations and interactions and make predictions. BD in another definition is “any voluminous amount of structured, semi-structured or unstructured data that have the potential to be mined for information” [3]. BD is characterized by the three V's: Volume, Variety and Velocity. In addition, BD also has Variability, Veracity and Complexity.

Volume is the most important characteristic of BD. The volume of data is growing exponentially. For example, in 2009 the world's total data volume was approximately 1.5 zettabytes (1 zettabyte is 1000 terabytes or 10^18^ gigabytes). In 2015, the data volume grew to 8 zettabytes and it is predicted that by 2020 it will be 44 zettabytes [16]. Biomedical data have contributed substantially to this overall growth in volume due to data-rich technologies such as various imaging modalities and the various omics (genomics, proteomics, etc.).

Variety or diversity of data is another characteristic as well as the main challenge of BD. The overwhelming majority of data including data in the biomedical literature is in an unstructured format containing text, images, multimedia, among others.Scientific articles are typical examples of unstructured data in that they do not have a predefined data model, as they are not organized in a predefined manner. They include raw text, images, videos, physiological and pathological data, among others.Such unstructured data are very difficult to understand using traditional programs due to irregularities and ambiguities. A combination of text mining, image, nucleotide and/or amino acid sequence analyses and other preprocessing steps are needed to give structure to this raw data and to extract the information or generate quantitative signature vectors. The most challenging is text preprocessing, requiring statistical parsing, computational linguistics and/or ML [17] to generate numerical summaries.

Velocity data have temporal dimension, are data in motion and is the third main characteristic of BD. Velocity means that the data collected can vary from a single batch/sampling, for example, the selected experimental end point through periodic sampling, in other words, multiple time points, through near real-time collection to real-time streaming data. An example of near real-time or real-time data collection is neurointensive care monitoring. The importance of such continuous data collection is obvious as it can provide the clinicians with trends, such as improving or worsening conditions over time. As the costs of collecting and storing data are getting less expensive, near real-time or real-time data streaming is becoming increasingly common.

In addition, BD also has Variability, Veracity and Complexity. Variability differs from variety in that it refers to the absence of uniformity. For example, a parameter that is expected to be the same can vary due to human or machine error. Variability can have substantial impacts on the reliability of data, in other words, how representative each data point really is, which in turn will affect data homogeneity. Veracity means that the data are uneven in quality, incomplete, ambiguous or deceptive. Filtering out inaccurate data is a serious challenge as it can lead to the classic ‘garbage in, garbage out’ scenario. Complexity is generally defined as many different components that interact with each other in multiple ways causing a higher order organization that is greater than the sum of its parts.

Incompleteness of data represents an especially serious challenge of BD approaches in biomedical research, including TBI research. Publications represent only a fraction of total data collected and accumulated during experimental TBI work or clinical studies, and these data are ‘curated’. According to conservative estimates, some 50% of the data from experimental and or clinical TBI research is never published for various reasons including failure of the experimental data to support the hypothesis. In addition to the unpublished data, which may be available in digital format, large proportions of ‘dark data’ contain laboratory notes, clinical notes, animal care records, among others.These data may reside on paper, analog media and/or on personal hard drives, and are thus called the ‘file-drawer phenomenon’ [8].

Although it appears counterintuitive, large volumes of incomplete messy data are more valuable and enable higher probability of correlation than clean, curated small datasets that may or may not be representative and almost certainly biased. One can call it the IBM versus Google approach for creating language translation. IBM fed the French and the English versions of Canadian parliamentary transcripts – small selected curated samples – into its machines to infer which French word is the best equivalent of the English word. The huge undertaking became stuck in the complexities of mathematical probabilities. The Google approach has been different. Google collected all available data from the Internet, an arguably messy task, and an incomplete and inaccurate source of languages, as opposed to the clean but small IBM sampling. Google has taken in billions and billions of pages from all kinds of sources and documents in multiple languages. The result speaks for itself: Google Translate currently covers 104 languages and the quality of the translations is fairly accurate. We should note here that IBM is catching up with Google as IBM's Watson cognitive computing technology can analyze BD to identify novel drug targets, among other biomedical applications [5].

## Traumatic brain injury

TBI is a spectrum disease. The severity of the impact ranges from severe to mild, with the latter also called concussion [18]. TBI accounts for approximately 30% of deaths caused by injury among young people under age 45, and it is the single most common cause of death and permanent disability in this group [19]. The incidence of TBI is staggering. In 2015, approximately 2 million individuals suffered with TBIs in the USA alone, and the number worldwide was approximately 60 million. The medical, economical and social expenses directly related to TBI are approximately 96 billion dollars annually in the USA alone. Injuries that include TBI cause the deaths of approximately 150 people per day in the USA resulting in approximately 50,000 deaths per year. The incidence of TBI has been steadily increasing and the number of TBI cases nearly doubled from 2001 to 2010 from 521 to 824 per 100,000 people in the USA [1]. The WHO has predicted that by 2020, TBI will be among the top three diseases causing death and disability [20]. As indicated by the difference between the rate of increase in emergency department visits versus hospitalization (70 vs 11%), the rise is mostly due to the surge in mild TBI/concussion cases [2]. Due to lack of uniformity in reporting requirements, there are controversies regarding changes in mortality [21]. However, mortality decreased substantially at locations with improved neurocritical care [22]. Severe and moderate forms of TBI increase the risk of Alzheimer's disease 2.3- to 4.5-times [23], and consequently multiply the already staggering medical, fiscal and social expenses related to TBI. At the other end of the severity scale, mild TBI/concussion accounts for approximately 85% of all TBI cases [2,24]. Mild TBI, especially when repetitive in nature, increases the risk for developing neurodegenerative conditions, such as chronic traumatic encephalopathy three- to five-times, thereby further increasing the disease-associated expenses [24].

The physical impact results in the primary injury process, structural and functional damage that is instantaneous and cannot be treated but only prevented by avoiding TBI. Based on the type of physical forces and how they interact with the head/brain, the primary injury process includes damage to axons, blood vessels, neurons and glia, and triggers the highly complex and dynamically changing secondary injury process [25].

There appears to be a correlation between physical forces and the secondary injury process. Mild TBI or concussion predominantly causes transient metabolic changes; whereas, severe acceleration/deceleration in TBI results in vascular [26] and axonal [27] injuries followed by complex downstream processes including inflammation [28]. Blast-induced TBI appears to have unique pathophysiology [29–31]. However, the exact correlation between physical forces and the biological response is not known. Moreover, the biological responses change over time, so the temporal aspect of these changes dramatically increases the data to be measured, monitored, collected, stored and analyzed. The cellular, molecular and structural changes associated with the primary (physical) and secondary (biological) responses to the injury manifest in functional changes observed clinically. These changes include a whole array of altered physiological responses, for example, decreased cerebral perfusion, depressed glucose metabolism, altered water balance, edema, among others, and neurobehavioral changes ranging from dizziness, confusion and memory impairment to loss of consciousness [32]. These clinically observed signs and symptoms change over time post TBI leading to the ever-increasing amounts of clinical data.

## Current use of BD in TBI

Efforts to improve the clinical practice guidelines to assess the severity of concussion have resulted in the development of several algorithms to evaluate changes in physical, cognitive, behavioral, imaging and neuropsychological levels [33]. Traditionally, the use of BD in concussion research has incorporated clinical guidelines that include multimodal subjective features, thereby producing significant challenges for clinicians attempting to diagnose concussion and the severity of the injury [33,34]. Collecting biological data that include functional testing results and blood biomarker analyses in combination with the collection of physical data that describe frequency, location and force of impacts will provide complex information at multiple levels, thereby generating massive amounts of data. Managing and sharing these data have led to efforts to improve sharing and distribution of BD within the TBI field. In response to the explosion in the amount of TBI data, numerous models for database repositories have been proposed and some have been established. Building upon a foundation created in 2009 by the International Mission for Prognosis and Analysis of Clinical Trials (IMPACT), an initiative to establish CDEs for TBI, was launched to standardize data collection across clinical trial sites. Data included demographics, clinical care, genetic and proteomic markers, neuroimaging and outcome measures to represent a range of TBI data including data relevant to all TBI studies, highly heterogeneous datasets and measures for which no consensus or validation has been achieved [35,36]. Importantly, the database contains few imaging data and virtually no monitoring data, thus greatly limiting the use of the database for BDA. Transforming Research and Clinical Knowledge in TBI multicenter prospective observational studies were then conducted to validate the feasibility of implementing CDEs among 650 subjects who received CT scans in the emergency room within 24 h of injury from level I trauma centers and one rehabilitation center in the USA [36]. Currently, Transforming Research and Clinical Knowledge in TBI houses data on 3000 patients from 11 sites in the USA and was the first to populate the Federal Interagency TBI Research (FITBIR) informatics system. Collaborative efforts between the National Institute of Neurological Disorders and Stroke and the Department of Defense created a national resource for archiving and sharing clinical research data on TBI [37]. The goals of FITBIR informatics system are to promote data sharing in the field of TBI, enable data sharing among individual laboratories and encourage connectivity with other platforms [38]. FITBIR currently stores over 200,000 data records that include detailed demographics, outcome assessments, imaging and biomarkers. By implementing the comprehensive interagency CDEs for TBI research as defined by the CDE work group, FITBIR provides tools and resources to extend the data dictionary. In this platform, qualified researchers can gain access to the data in the hopes that novel modeling approaches may uncover relationships not realized by the original data collectors, thereby leading to additional studies and successful clinical trials for treatment of TBI. The CDE initiatives for clinical as well as preclinical TBI studies are giant steps toward improving disease severity classification, unifying data entry, depositing and archiving data. The success of the initiative is reflected in the increasing entries as well as analyses and studies using FITBIR.

Neurointensive care units (NICU) generate very large volumes of data collected during continuous monitoring of vitals, physiological and biochemical parameters such as cerebral perfusion pressure, cerebral blood flow, brain tissue oxygenation, intracranial pressure, changes in intracranial glucose metabolism, among others[32,39]. Combined with the outputs of various imaging modalities, EEGs and other diagnostic monitoring, each patient generates staggering amounts of data during the NICU stay. However, there is currently no unified protocol for analyzing data to help in developing guidelines for the patient's management, and in the absence of follow-ups, the correlation between early disease management and long-term outcomes cannot be established [40]. While neurointensive monitoring generates digital and relatively simple datasets, neuroimaging, probably the most powerful diagnostic tool in NICU, produces huge amounts of very complex data. The absence of standardized timing of image acquisitions, lack of uniformed imaging protocols and other unresolved issues make the BD approach in the NICU rather challenging [41].

In this context, Smith *et al*. created a defined set of CDEs for use in preclinical models that consisted of ten modules divided into a Core Module with 57 CDEs and Injury-Model-Specific modules for nongeneralizable elements [42]. Among the Core CDEs, CDE domains included animal characteristics, animal history, assessments and outcomes and injury model characteristics. Within the Injury-Model-Specific modules, categories included weight drop, fluid percussion, blast, penetrating ballistic-like, hemorrhage, increased intracranial pressure and porcine rotational acceleration. Taken together, development of preclinical CDEs promotes the use of a common language among researchers using animal models of TBI thereby facilitating ease of cross comparison among studies.

## Future of BD in TBI

**Figure 1.**
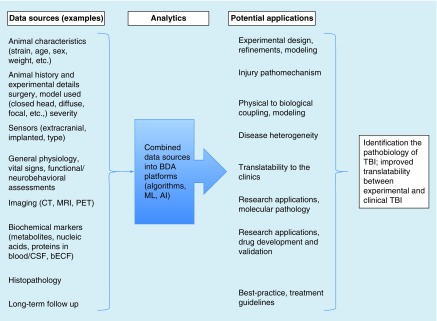
**Overview of potential Big Data Analytics approaches in experimental traumatic brain injury.** Examples of data sources (see also [42]) and potential application using BDA approaches that can improve modeling, understanding the pathobiology and translatability between experimental and clinical TBI. AI: Artificial Intelligence; BDA: Big Data Analytics; bECF: Brain extracellular fluid; CSF: Cerebrospinal fluid; CT: Computer-assisted tomography; ML: Machine Learning; TBI: Traumatic brain injury.

**Figure 2.**
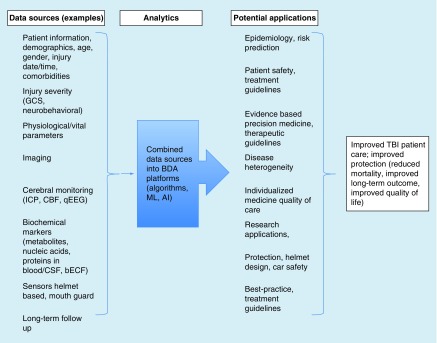
**Overview of potential Big Data Analytics approaches in clinical traumatic brain injury.** Examples of data sources (see also [43,44]) and potential application using BDA approaches that can result in improved patient care, reduced mortality and better postinjury quality of life. AI: Artificial Intelligence; BDA: Big Data Analytics; bECF: Brain extracellular fluid; CBF: Cerebral blood flow; CSF: Cerebrospinal fluid; GCS: Glasgow Coma Scale; ICP: Intracranial pressure; ML: Machine learning; qEEG: Quantitative electroencephalography; TBI: Traumatic brain injury.

The simultaneous increases in data availability and analytical capabilities create a golden opportunity to use BDA in TBI. We have illustrated some of the potential data sources for BDA in experimental (Table 1) and clinical TBI (Table 2). The data sources represent CDEs recommended by National Institute of Neurological Disorders and Stroke's Expert Panel for preclinical [42] and clinical TBI [43], respectively. Of the many CDEs detailed in these publications, we have selected eight potential data sources and listed their strengths and current limitations. Figures 1 and 2 list some of the potential applications of and benefits resulting from using the BDA approach in experimental and clinical TBI, respectively. From the eight listed data sources, we discuss three in more detail: sensors, imaging and biochemical markers. These sources were selected because they have high translational relevance between experimental and clinical TBI studies and provide quantifiable and structured data ideal for BDA approaches. Combining and analyzing BD even from these three sources would provide important correlations and better understanding of the relationship between physical forces and biological outcomes, in other words, the pathological changes triggered by the insult. Time-dependent sampling of these outcomes, imaging and biochemical markers can indicate disease progression and significantly improve predictions. Developing predictive models would result in substantial savings in healthcare costs and would improve patient care.

**Table 1.** **Some potential data sources for Big Data Analytics in experimental traumatic brain injury.**

**Source**	**Description**	**Strengths**	**Limitations**
Animal characteristics	Species, age, sex, weight	Homogeneous population, reproducibility	Gender and age biased (mostly young males used), translational value is an issue, unstructured data

Animal history and injury model	Experimental details, surgery, modeling (closed, open, rotational, focal), etc. severity (physical parameters)	Reproducible, set and quantifiable physical parameters	Mostly small rodents used, scalability (anatomy, physiology) to human is a major issue, unstructured data

Sensors	Extracranial or implanted	Quantitative, 3D distribution of actual *g*-forces	Extracranial sensors are not frequently used in animal studies, implanted sensor data are challenging to translate into clinical use

General physiology, vital signs, neurobehavioral assessments	Indicate injury-induced changes in physiological parameters, (heart rate, blood oxygenation, etc.) and in specific neurobehavioral functions (learning, memory, anxiety, etc.)	Objective measures of changes in physiology, structured data, specific functional impairments and translational relevance	Physiological monitoring is rarely used in experimental TBI, neurobehavioral data are investigator dependent and unstructured

Imaging	Various modalities (CT, MRI, PET)	Clinically relevant, repeatable, noninvasive, provides morphological (molecular PET) information	Rarely performed in experimental TBI, no standardized analysis programs, difficulties comparing data from different laboratories, very large data

Biochemical markers	Injury-induced changes in serum or CSF (or bECF) levels of metabolites, nucleic acids and proteins	Can identify the molecular pathology of the injury process, can identify targets for therapeutics. Quantitative, structured data	Too many candidate biomarkers, no consensus, no clear association between biomarker values and injury and outcomes, not widely available

Histopathology	Standard histology and immunohistochemistry	Identifies brain regions affected by injury, provides cellular and molecular level of information about the pathobiology	Terminal stage, difficulty in translating to clinical outcome measures; unstructured data, variability among laboratories

Long-term follow-up	Neurobehavioral testing at late postinjury time points	Assessing disease progression and/or the efficacy of therapeutic interventions	Rarely performed in animal studies, the correlation between rodent and human physiology and timelines are not well understood

bECF: Brain extracellular fluid; CSF: Cerebrospinal fluid; CT: Computer-assisted tomography; *g*-force: Gravitational force; TBI: Traumatic brain injury.

**Table 2.** **Some potential data sources for Big Data Analytics in clinical traumatic brain injury.**

**Source**	**Description**	**Strengths**	**Limitations**
Patient information	Patient demographics (age, gender, etc.), comorbidities, medications, injury date and time	Diverse data reflecting the actual medical records, previous conditions	Not always available electronically, or in timely manner, mixture of structured and unstructured data

Injury severity, functional impairment	GCS, LOC, etc., assess key neurological and behavioral functions, indicators of severity	GCS, LOC, widely used, international standard of assessing functional impairment, numeric output	Subjective, not useful in mild TBI/concussion, multiple pathologies can lead to identical GCS score

Physiological/vital parameters	Injury-induced changes in key physiological parameters (heart rate, blood oxygenation, etc.)	Standardized outputs, structured data	At present, data are not universally stored and available for (meta)analysis; co-morbidities (polytrauma) can majorly affect data

Imaging	Imaging data from CT, MRI, PET	Routine technology and consistency of CT, increasing usage of MRI and its various modalities	Data quality is uneven, variability in data types, atlases and interpretations, user specific

Cerebral monitoring	ICP, CBF, qEEG provide quantitative data about intracranial physiology and brain activity	Standardized, numeric, structured data, real-time or near real-time dataflow	Data quality is uneven, variability in data types, variability among users

Biochemical markers	Protein or metabolic data in serum or CSF (or bECF) samples	Can potentially inform about the secondary injury process, can guide therapy	Many candidates, no verified marker, assays are not widely performed, presence of structured and unstructured data, user specific

Sensors	Helmet or mouthguard providing physical data	Reflect the actual cranial, 3D distribution of *g*-forces, real time dataflow, quantitative	Not standardized, multiple types, the relationship between *g*-forces and biological outcome needs to be established

Long-term follow-up	GOS, WAIS, SWLS, DRS, FIM, etc., measure wide range of neurobehavioral and quality-of-life outcomes	Multiple time points enable monitoring disease progression, assessing treatment efficacy	Not universally performed, expensive, needs dedicated staff

bECF: Brain extracellular fluid; CBF: Cerebral blood flow; CSF: Cerebrospinal fluid; CT: Computer-assisted tomography; DRS: Disability Rating Scale; FIM: Functional Independence Measure; GCS: Glasgow Coma Scale; *g*-force: Gravitational force; GOS: Glasgow outcome scale; ICP: Intracranial pressure; LOC: Loss of consciousness; qEEG: Quantitative electroencephalography; SWLS: Satisfaction with Life Scale; TBI: Traumatic brain injury; WAIS: Wechsler Adult Intelligence Scale.

## Sensors

Because TBI is caused by physical forces that can be measured and quantified, an important step toward using BD approaches and developing a TBI dosimetry is to understand the correlation between the physical forces and the biological response [45]. The distribution of *g*-forces, focal or diffuse impacts along with their intensities directly delivered to the head, generates highly complex biomechanical responses to the physical forces. Analyzing the physical parameters of the impact as a function of the biological response can provide critical data about the physical and biological threshold for injury severity and pathomechanisms [46,47]. In the absence of outward signs of brain injury, tracking the frequency and severities of head impacts currently relies heavily on self-report or film review and is therefore estimated at best [48,49]. Moreover, gauging the force from an impact is not possible using these reporting approaches. Implementing methods to lessen injury associated with TBI is dependent upon tracking accurate measurements of forces transmitted to the head. Thus, approaches to track and gauge the cumulative effects of repeated mild TBI are at the forefront of investigation. Understanding the relationships among frequency, location, force and thresholds for concussion with those of acute and long-term changes in physiology, cognition, vision, balance and presence of blood markers is hindered by accuracy of recording impacts sustained. Adding to this already complex landscape, the use of instrumented accelerometers has significantly increased the ‘long tail’ data in neuroscience [8]. In fact, many organizations employ instrumented accelerometers placed in helmets, on caps or headbands worn under the helmet or embedded in mouth guards [50–52]. Tracking the numbers, location and force with which an impact is sustained for individuals generates massive amounts of data. For example, in one study, the maximum number of head impacts for a single player was 1444 for the season [53] illustrating the vast amount of data that can be collected using instrumented accelerometers. Capabilities of such recordings include information regarding the numbers of hits, location of the impact, linear and rotational *g*-force, and allow for theoretical thresholds to be set to gauge force required for concussion and measurable changes in other output modes such as vision, balance, cognition, among others.

Numerous studies have been conducted using the Head Impact Telemetry (HIT) system that allows for comparisons of numbers, force and location of impacts with acute clinical outcome of symptomology, neurocognitive performance, balance and others [53–64]. As illustrated by these studies, the amounts of data generated by this recording system are vast and diverse. Objectives of studies range from attempting to set *g*-force thresholds for accurate diagnosis of a concussion, to the location of impact on the head as a diagnostic factor, to the relationship between magnitude of impact and postural control. Recordings from one study using the HIT system in 72 collegiate football players, recorded over 57,000 head hits, each with a specific location and linear versus rotational acceleration [55]. Other studies aimed to translate the number of hits and thresholds of 60 *g* (low) and 90 *g* (high) into changes in neurocognitive function and balance performance [54]. Studies conducted by Greenwald *et al*. collected data from over 289,000 individual impacts [57]. In these studies, 17 concussions were diagnosed from which a single impact was identified as the concussive event. Tracking events that may have led up to or contributed to the concussion is made possible with instrumented systems [57]. Broglio *et al*. tracked all head impacts in 78 high school football players and recorded 54,247 individual impacts [65]. These studies report that rotational acceleration, linear acceleration (>96.1 *g*) and location (front, top and back) yielded the highest predictive values for concussion. Results from earlier studies suggested that the HIT system proved effective to collect real-time impact events that could be combined with clinical evaluations [66].

Results from several studies utilizing mouthguard-embedded accelerometers also illustrate the complexity and volume of data that are collected in investigations. In one study conducted with rugby players, a total of 20,687 head impacts >10 *g* and up to 106 *g* were recorded from 38 players over 379 player match hours during the season yielding a mean of 564 ± 618 head impacts per player, each with unique linear and rotational kinematic parameters associated per hit [52]. In another set of studies, attempts to associate the number of hits, and linear and rotational forces with clinical and cognitive changes and investigated if subconcussive impacts during preseason football practice in collegiate football players caused changes in near point of convergence or symptom scores using the Sports Concussion Assessment Tool 3 [48]. This study stratified players into low- and high-impact groups based on the frequency and linear and rotational forces with which they were hit. Results indicated that in the high-impact group there was a linear increase in near point of convergence over time that resolved post season whereas, no changes were detected in the low-impact group [67]. No changes were observed in the Sports Concussion Assessment Tool 3 symptom scores in either group supporting the need for multilevel testing approaches. Collectively, results from these studies highlight the scale and diverse types of data that can be collected and analyzed using instrumented systems to track impacts and thus emphasize the need for streamlining, management and sharing of pre-existing common data elements and interpretation of variables.

## Biochemical markers

The presence of brain-derived proteins in the blood or cerebrospinal fluid (CSF) can provide objective measures for determining TBI's severity, identifying the pathomechanisms of the secondary injury process and predicting recovery [29,44,68–71]. There is a vast literature reporting changes in candidate TBI biomarkers in both small and large cohort studies. The paradox of blood-based protein biomarkers in TBI is that there are fairly well-established biomarkers for severe TBI [72] where other diagnostics, primarily imaging also provides detailed information about the type and severity of injury. In mild TBI/concussion where imaging is negative, there is a substantial need for blood- or CSF-based biomarkers [71,73]. Also, even though current blood-based biomarkers can indicate the extent of damage, they do not provide information about the pathological changes of the secondary injury process, and thus they cannot identify therapeutic targets or help with evidence-based therapy.

Currently, two approaches are used in the discovery of potential biomarkers: the top-down and bottom-up methods [74,75]. Top-down approaches involve consideration of a disease process and hypotheses are formed and tested, often resulting in bias and low throughput efficiency [74]. On the other hand, bottom-up approaches utilize high throughput -omics modeling, whereby unbiased quantification of all molecules of a specific type (e.g., cytokines) results in large lists of potential candidates. These two methodologies are limited in their capacity to provide novel connections among complex molecular events associated with TBI and rely on previously identified interactions. However, information provided by both approaches may be combined into a data repository format where systems biology networks can provide yet another level of integration of our knowledge base [69]. For example, as explained by Feala *et al*., TBI high-throughput data consisting of canonical pathways and protein–protein interaction maps can be integrated to identify TBI-specific pathways and protein interactions [74]. In this model, beginning from a list of condition-specific (e.g., severity level or post-TBI time point) high-throughput-omics data, mapping these hits onto pathways and protein–protein interaction scaffolds would allow for determination of patterns related to injury-specific responses [74]. Importantly, this systems biology approach may contribute to refining some of the emerging CDEs as our understanding of the global picture of TBI expands [36]. In response to these challenges, Yue *et al*. conducted a prospective multicenter observational study to validate the feasibility of applying CDEs for TBI. In a study by Dabek and Caban, a framework was presented, whereby longitudinal data from nearly 100,000 concussion patients were utilized to build a predictive model of the likelihood of developing a psychological disorder within the first year post-TBI [34]. Using post-traumatic stress disorder as an example, the model validated 16,045 patients from among 89,840 service members with over 5 million clinical encounters with an accuracy of 85% (86.52% area under the curve [AUC]) for developing this condition during the first year post-TBI.

Changes in levels of many potential blood biomarkers have been proposed to correlate with brain injury, but differences in study design and interpretation have made it difficult to validate TBI-specific markers. Although numerous blood biomarkers are under intense investigation, to date there are no US FDA approved biomarkers for brain injury. One problem with identifying a suitable biomarker has been the sensitivity and specificity for TBI. Diversity across cohort-based studies impedes the utility of large database repositories in making diagnoses based on blood marker changes reported in the literature. In this context, some studies propose to combine measures from several biomarkers to gain more clear insight into changes in biological processes post-TBI [69,76]. In line with these studies, FITBIR outlines detailed procedures for blood and CSF collection post-TBI to promote standardization and decrease variations in experimental approaches. Other questions surrounding the choice of a particular blood biomarker relate to cellular origin, normal and pathological function, and possible reasons for changes in blood levels [77]. In fact, in a study examining the magnitude of the effect sizes of biomarkers, results showed that highly cited biomarker studies many times report larger effect estimates for hypothesized associations than are supported by meta-analyses evaluating these associations [78]. In addition, given the complex nature of primary and secondary injury components in TBI, biomarker development has added limitations in specificity, as TBI shares many neuropathological features, such as inflammation and cellular damage with other CNS diseases and disorders.

Despite current problems with biochemical markers, some of them listed above, injury-induced changes in biochemical marker levels in various biofluids, blood or CSF are ideal data sources for BDA approaches for several reasons. The data are numeric, structured, frequently measured at various post-injury time points, collected in both experimental and in clinical TBI studies, recorded and archived. When and where they are measured, important additional clinical or experimental data such as injury severity, the extent of functional impairments, imaging, among others, are also available enabling the use of BDA to analyze biomarker data in the context of other data sources.

## Imaging

With significant and rapid advances in the field, including the technology to acquire various imaging modalities coupled with high-speed image processing and analytics programs, *in vivo* imaging represents a perfect example of promises and challenges of using the BDA approach in TBI research, diagnosis and management.

Due to their data-rich nature, *in vivo* imaging represents unique challenges because as technologies improve so do the increases in the amount of data generated. As of today, just the acquired neuroimaging data alone are an average of 20 GB per published study [79]. This amount of data is only the tip of the iceberg as publications may or may not include aggregated and annotated data generated by using BrainMap, BrainSpell or other tools. This enriched data enable re-analysis, data mining and meta-analyses. The amount of data increases substantially when unthresholded statistical maps are also deposited allowing re-use and complete re-analyses, both of which are important requirements for BDA approaches. While a single study of this size does not pose difficulties for processing and analysis, large numbers of datasets required for a successful BDA approach pose significant challenges. Currently, an fMRI scan can obtain multiple BOLD image volumes of the whole human head per second and advanced diffusion tensor imaging (DTI) imaging is capable of resolving 512 or more fiber directions resulting in incredibly fine resolution and huge volumes of data [80]. These and other technical improvements mean that the amount of data is doubling in roughly every 2 years. To illustrate some of the issues with the amount of data imaging generates, the Human Connectome Project's first dataset is approximately 18 terabytes, which is currently available on multiple hard drives delivered by mail [79]. Probably, the most advanced imaging repositories have been established for Alzheimer's disease (Alzheimer's Disease Network Initiative) [81–83]. However, the nature of the disease is substantially different from TBI, so it may not serve as a good example for TBI. FITBIR [37] and StrokeNet [11] are two interrelated imaging repositories for BDA approaches. However, in the context of TBI, the lack of validation of many techniques in large cohorts using consistent methods for retrieval and analyses of data yields inferential conclusions. The absence of truly normative data also creates a significant obstacle in identifying specific changes, especially in cases of mild to moderate injuries [84,85]. During a ‘Joint ASNR-ACR-HII-ASFNR TBI Workshop: Bringing Advanced Neuroimaging for TBI into the Clinic’ to reach agreement on recommendations for creating a normative database, the committee recommended streamlining collection, phenotypic and outcomes data to allow sharing and queries across platforms by using CDEs [86]. Head computed tomography, MRI including T1- and T2-weighted, fluid attenuated inversion recovery, diffusion- or susceptibility-weighted sequences can detect acute intracranial sequelae and chronic effects of TBI, but methods outside of these more standard approaches lack validation for milder forms of TBI and are therefore, not established for clinical use at the individual level [11,84].

There are substantial challenges at the technical level for imaging in TBI. The main challenge is standardization or how to take into account differences between various laboratories using different acquisition rates, resolutions, scanning parameters, among others[79]. Even identical scanners used at different locations can generate differences in the quality of primary data, which in combination with different atlases and analytical programs make large-scale comparative studies challenging. BDA approaches will play a major role in establishing such a model by combining incoming high fidelity imaging data that includes white matter connectivity and functional activity in addition to basic anatomical information. The amount of data from TBI studies will increase exponentially as more and more institutions are using scanners at increasing frequency. Analyzing imaging data to find correlations between structural and molecular changes (biomarkers) and neurobehavioral outcomes represents a serious challenge due to the size and varying structures of data.

## Cellular biomechanics, *in vitro* & *in vivo* modeling

Connecting cellular biomechanics data to data derived from animal modeling and applying the combined knowledge to clinical TBI would substantially increase our understanding about the physical to biological coupling down to the molecular level, which would guide evidence-based therapies. The physical forces encountered by individual cells will determine survival or death, and in the case of survival, it initiates a complex molecular response to recover and regenerate. In contrast to other systems, such as the vascular system, which is constantly exposed to mechanical loading, stretch during the cardiovascular cycle, neurons and glia are mechanically naive and protected. Also, the forces of the mechanical insults are extremely high-velocity events in contrast to the systolic/diastolic cycle [87].

Cell culture models [87–89] in combination with various outcome measures have provided critical insights into cellular and subcellular responses to mechanical forces. These works identified several structures termed ‘mechanosensors’ that include the voltage-gated sodium channel, or NMDA receptor that responds to the mechanical forces induced by cell deformation with altered channel activation [87]. Data from these experiments are used to build *in silico* models of TBI [90–92]. However, the human brain contains approximately 100 billion neurons and ten-times more glial cells. Thus, high-fidelity modeling of TBI that includes detailed molecular responses to mechanical forces cannot be accomplished without considerable use of BDA approaches.

From the biomechanical perspective, the cranium and the intracranial structures such as the dura and pia mater, and the trabeculae provide the first line of defense in mitigating the physical impact and have distinct tissue properties from the cerebrum and cerebellum proper, each responding to the physical impact in different ways [93–95]. The biomechanical properties and responses to physical/kinetic forces of the various anatomical structures, tissue types have been measured, analyzed and the data are available for re-analysis and integration with other, for example biochemical data. Boundaries or intracranial ‘fault lines’ exist between different anatomical structures with different material properties, brain tissue versus blood vessels, gray versus white matter or brain tissue versus CSF. Such fault lines exist at the cellular and subcellular levels due to different material properties, elasticity, compressibility of axons versus cell bodies; capillaries, endothelial cells versus astroglial foot processes; myelin sheaths versus axons. These fault lines are the anatomical substrates for the vascular and axonal injuries triggered by the physical insult [96–98]. Neuroanatomy (e.g., the directionality of white matter tracks) greatly modifies and determines the extent of damage caused by similar *g*-forces. Accordingly, in addition to the actual *g*-forces, the 3D distribution of the *g*-force is critical in determining the biological response to the mechanical forces further increasing the number of data elements. *In vitro* modeling using cell and tissue culture systems have generated substantial amounts of data at the cellular and subcellular levels about the biomechanical/biological responses as functions of physical forces [88,99–101].

One of the greatest challenges in TBI is to improve translatability of experimental data into clinical practice. There have been numerous animal models developed over the last several decades that have attempted to mimic clinically observed conditions. They are categorized as focal, diffuse, penetrating, blast, among otherstypes of injuries with each focusing on a specific type of physical impact. Animal modeling of TBI including issues with current models has been recently reviewed in an excellent book chapter [102] and is beyond the scope of this review. Animal modeling of TBI has resulted in 3000+ publications containing mostly unstructured data. Roughly 90% of the models are using rodents that have lissencephalic brains. In addition, basic biology, physiology and pathobiology of rodents are significantly different from humans, so without employing BDA approaches, the correlation and relevance between experimental findings and clinical cases remain rather subjective guesswork. These issues are known but are easily addressable gaps in outcome measures between experimental and clinical TBI research. The physical forces are known for *in vivo* TBI studies but, without the BD approach, we currently lack the ability to combine *in vitro* and *in vivo* data aimed to identify the pathobiology of TBI at the cellular, subcellular and molecular levels.

## Legacy data

CDE and FITBIR are important for current and future TBI studies, but what can be done about the TBI legacy data accumulated over decades? Full implementation of CDE is not yet in sight. Moreover, it would take enormous amounts of time, effort and funding to load existing, unstructured data into traditional relational databases (such as FITBIR).

Powerful tools using BD and BDA approaches are available and they have been successfully employed in counter-terrorism efforts, fighting crime, bank fraud, among others. Palantir [103] and Ayasdi [104] have developed BD Analysis software capabilities and ML/AI systems. Tools developed by these and other companies, such as IBM provide predictions, relationships and correlations by analyzing giant, messy, unstructured incomplete datasets. Think of TBI/concussion as a massive, complicated intelligence game where important facts are hidden in huge amounts of irrelevant and unstructured data. Tools such as those developed and used by, for example, Palantir Technologies can uncover terrorist networks and plans to prevent attacks using far less reliable, more fragmented, less structured information available to the intelligence community than is available to the TBI community. Using similar tools may uncover correlations and connections addressing some of the TBI field's most pressing issues.

Similar to other fields, a potential solution for BDA in TBI is to aggregate the existing raw data along with extended metadata in a ‘data lake’ and use Palantir's or Ayasdi's or IBM's algorithms, ML/AI capabilities to identify repeatable patterns, relationships and correlations [5]. A data lake is a storage repository that contains a huge amount of raw data in its native, unstructured format in so-called flat (nonhierarchical) architecture. Data elements are tagged with a unique identifier and extended metadata tags. Such a data lake can be queried then with a specific question and a smaller dataset can be analyzed to answer a specific question.

A typical article in a scientific journal, with images, is in the megabyte size range and the entire published TBI literature is in the terabyte/petabyte range, but the published data represents only a fraction of data collected during the study. From the BD perspective, in addition to their unstructured formats, there are other issues with the published TBI or virtually any other biomedical literature [7,12–13]. They are part of the so-called ‘long-tail’ data comprised of both published data and unpublished dark data [8]. Publications represent only a fraction of total data collected and accumulated during experimental TBI works or clinical studies, and they are curated. The current total TBI literature published in peer-reviewed journals is just over 33,000 papers. If we take into account the previously mentioned dark data, completed but unpublished works and additional information, the number is close to 100,000 in text heavy, unstructured data format. Current mining of this legacy data is performed manually using PubMed or similar search engines involving heavy use of the searcher's skills as well as judgments, and so this approach is prone to be subjective and biased.

## Challenges

Successful use of BDA approaches will require three critical changes in our current practices and thinking: collect and store all data; accept messiness of the data; and give up causation for correlation. It should be noted that employing BDA will originally reveal only correlations but not causations, but the accumulated correlations over time will allow establishment of causative relationships. A lot of evenly unstructured data with uneven quality is better than a small, curated dataset.

Experimental (preclinical) and clinical TBI studies use different outcome measures, methodologies, different species with different anatomy, physiology, different physical forces resulting in an enormous gap between the two fields. Leading examples are the use of the Glasgow Coma Scale, length of loss of consciousness, alterations in mental/conscious state and post-traumatic amnesia, Glasgow Outcome Scale in clinical TBI for triaging and selecting patients for clinical trials and assessing disease progression and clinical trials. There are somewhat similar functional assays such as Injury Severity Score, Neurological Severity Score, among others, available for rodents in experimental TBI, but we do not know how these correlate with the gold standard Glasgow Coma Scale and other clinical tests [105]. The resulting paradox is that we know the extent of the functional deficits, but not the parameters of the causative physical forces in clinical TBI; whereas, in experimental TBI, we know the parameters of the physical forces (we can calibrate them), but not functional deficits caused by these forces. The approach to collect both physical data using sensors as well as monitoring functional deficits and collecting biochemical data as outlined above will help to identify the correlation between physical impact and biological response. But the field badly needs BD approaches to establish the correlation between injury-induced changes, functional deficits, biochemical and structural changes detected in humans and in rodents using already available data. Only after we understand the correlation between injury-induced changes in clinical versus experimental TBI can we translate promising preclinical pharmacotherapies into clinical trials with high fidelity, and vice versa can we design more clinically relevant experimental studies.

As we collect more and more data, for example, from patient monitoring at NICUs [40] or physical, biological and clinical data from athletic fields following concussions, we will be able to understand correlations. In biology or in diseases such as TBI, most events are probabilistic rather than certain. Imagine, for example, if all NICUs collected and stored all the functional, imaging and biochemical, and similiar data from all patients [40]. Or, all the physical, biochemical, imaging and functional data related to concussion were collected at the sidelines of athletic fields. Or, if in experimental TBI, animals were being monitored for outcome measures mirroring clinical practices and all of the data were collected and stored along with unique experimental data such as histopathology [105]. Using BDA would enable us to use the collected BD to discover correlations between, for example, altered cerebral perfusion pressure, cerebral blood flow, cerebral metabolic rate of oxygen, cerebral metabolic rate of glucose consumption and long-term functional outcome; or to discover correlations among *g*-force, directionality, biochemical changes, functional impairments and long-term outcome; or to discover correlations between biochemical and functional outcome measures and cellular and molecular levels of pathological changes bringing experimental TBI studies closer to clinical needs. However, we must accept that the collected data will be incomplete and messy. But, large volumes of incomplete, messy data are more valuable and enable higher probability of correlation than clean, curated small datasets that may or may not be representative.

Maybe the greatest challenge for us as TBI researchers is to change our habits. Such changes should include recording and storing everything digitally so we can reduce the amount of dark data. We must make all the data available for (re) analysis. The increasing use of CDEs will enable us to fill FITBIR with structured data that is relatively easy to analyze. The reality is that due to the nature of the disease, TBI/concussion is the result of accidents, so only a fraction of critical information will be deposited according to standards (CDE). This is especially true for mild TBI, where the individual may or may not be taken to the ER, and may or may not be seen by a concussion specialist. Accordingly, most of the data will remain incomplete, unformatted, fragmented and unstructured so we need to seriously think about employing Palantir, Ayasdi, etc., type massive BDA approaches to analyze existing data and also incoming clinical data. On the experimental side, there is a goldmine of published legacy data available, also unstructured, messy and incomplete and only BDA can help to find the correlations we so much need to improve protection and patient care.

## Future perspective

BD and BDA will revolutionize the TBI field within the next decade. The changes will be driven by the combination of increasingly powerful and capable BDA, and AI and ML (AI/ML) platforms coupled with financial incentives to use these technologies. One of the first fields transformed by implementing BDA and AI/ML approaches will be neurocritical care. BDA and AI/ML will enable us to find correlations between the dozens and dozens of streaming real-time data from physiological monitoring, imaging, biochemical and functional biomarkers. This new approach will transform the current practice of triaging, diagnostics, treatments and prognosis into highly integrated, evidence-based patient care. Because applying BDA and AI/ML technologies at NICU will result in significant savings in healthcare costs, insurance companies will provide the necessary financial incentives to implement these technologies. Another implementation of BDA and AI/ML will be (re) analyzing existing ‘legacy’ data. Only BDA and AI/ML approaches can help to establish new correlations using TBI legacy data that have accumulated over decades of TBI research. These tools will uncover new correlations and connections that will drive diagnostic and therapeutic Research and Development (R&D) efforts. Using BDA and AI/ML approaches will enable fast and efficient validation of experimental data ‘*in silico*’ which are much less expensive than repeating entire sets of preclinical and clinical studies. This will guide R&D efforts and eliminate unnecessary duplications. The availability of these approaches will motivate pharmaceuticals – which have withdrawn from the TBI R&D field [106] – to invest in the use of BDA and AI/ML because of the profit they can generate given the share size and potential of the TBI pharmacotherapy market. The third major change – quantum leap – in utilizing BDA and AI/ML in TBI will be in the establishment of predictive ‘dosimetry’. The physical forces will be measured and recorded by using the next generation of sensors. Streaming real-time data from sensors will generate a whole new level of data both in quantity and in quality. BDA and AI/ML will determine the correlations between physical forces and the biological response (the physical-to-biological coupling) using legacy data as well as actual imaging data with biochemical, neurobehavioral, among others monitoring. Establishing correlations between physical and biological data will help to develop better head protection and safety guidelines, and will also help to determine ‘safe return to play’ and ‘safe return to duty’. Such predictive TBI/concussion dosimetry will revolutionize entire industries ranging from automotive, sport/athletics, healthcare, elderly care, among others. These industries along with insurance companies will be investing massively in using BDA and AI/ML because of the enormous financial benefits derived from developing improved physical protection, reducing insurance claims and costs associated with healthcare, especially with elderly care. TBI R&D has reached its ‘strategic inflection point’, more of the same will not work as sadly illustrated by the 100% failure rate of clinical trials, BDA and AI/ML have the potential to put TBI R&D back into a new phase of potentially exponential growth. Welcome to the future!

**Executive summary**

**Traumatic brain injury**

- Traumatic brain injury (TBI) is the most heterogeneous and most complex among the disorders of the CNS.
- Mild TBI (concussion) is the most common type of TBI and when sustained repetitively increases the risk of developing neurodegenerative disorders.
- TBI is rapidly becoming one of the top three diseases causing death and disability worldwide.
- TBI is caused by wide ranges and types of physical forces.
- Biological responses to TBI occur at multiple levels including structural, physiological, behavioral and molecular that manifest in complex and dynamically changing clinical symptoms.
- The pathobiology of TBI is poorly understood, there are no accurate diagnostics and prognostics for TBI.
- There is no specific therapy for TBI, the failure rate of clinical trials is 100%.
- It appears that the current approach of analyzing small, representative, curated data will not be able to provide solutions to the complexity of the disease.

**Big Data**

- Big Data (BD) enables collecting, storing and analyzing voluminous amount of data.
- BD approaches can use unstructured, incomplete, irregular and ambiguous data as long as the dataset is large.
- Analyzing BD using Artificial Intelligence, Machine Learning and Cognitive Computing can identify new correlations not available by traditional approaches.
- BD has been successfully employed in fields of logistics, counter-terrorism efforts and healthcare.

**BD in TBI**

- BD is ideally suited for TBI where most of the data is unstructured, incomplete and messy.
- BD TBI requires collecting and storing all data elements, physical, biological, structural and clinical that may not be curated and may remain incomplete.
- Employing Big Data Analytics (BDA) in TBI can reveal correlations between physical forces collected by sensors, biological responses such as imaging, functional impairments, molecular pathologies and their temporal patterns.
- The accumulated correlations over time will allow establishing causative relationships.
- The Federal Interagency TBI Research and Common Data Elements initiatives provide the framework for TBI data deposition.

**Future of BD in TBI**

- In order to take advantage of BD, investigators should collect, store and make all data available for (re) analysis and reduce ‘dark data’ in TBI.
- Employ or customize existing BDA approaches such as Palantir or Ayasdi that have been proven successful in identifying correlations using similar complex data.
- An important proof of concept of such a BDA approach would be to establish correlations between experimental and clinical data.
